# Supplementary material for: Cancer Survivors’ Long-Term Health Service Costs in Queensland, Australia: Results of a Population-Level Data Linkage Study (Cos-Q)
Source: Int J Environ Res Public Health. 2022 Aug 2;19(15):9473. doi: 10.3390/ijerph19159473 (PMC9368477; doi:10.3390/ijerph19159473)
Supplement: Supplementary file 1 [file ijerph-19-09473-s001.zip › ijerph-1829806-supplementary.pdf]

Supplementary Material

Table S1. Mean annual Healthcare Costs (2013-16) in AU$ per Person by Age Group and Survivorship Status (Patient-level & Cohort-level)

Age group (years)	Alive			Deceased			Total cost					% of total cost
	N	Mean cost per person	SD	N	Mean cost per person	SD	N	%	Mean cost per person	SD	Sum of costs for cohort	
0-4	796	$24,197	63,547	24	$77,804	59,553	820	0.4	$ 25,766	64,041	$21,128,116	0.6
5-9	492	$22,287	42,571	16	$97,311	78,289	508	0.2	$ 24,651	45,918	$12,522,478	0.3
10-14	561	$20,993	46,980	16	$121,273	111,510	577	0.3	$ 23,774	52,356	$13,717,399	0.4
15-19	1,238	$13,958	39,867	27	$71,327	68,381	1,265	0.5	$ 15,183	41,478	$19,206,505	0.5
20-24	2,075	$8,865	28,658	43	$59,061	59,123	2,118	0.9	$ 9,884	30,399	$20,933,600	0.6
25-29	3,450	$8,168	18,546	79	$58,746	46,754	3,529	1.5	$ 9,300	20,990	$32,821,302	0.9
30-34	5,037	$8,645	19,033	142	$56,035	45,030	5,179	2.2	$ 9,944	21,620	$51,501,098	1.4
35-39	7,316	$9,388	18,757	240	$55,607	52,387	7,556	3.3	$ 10,856	22,207	$82,029,050	2.2
40-44	10,709	$10,386	20,259	443	$51,660	46,010	11,152	4.8	$ 12,026	23,303	$134,113,230	3.7
45-49	15,359	$10,437	19,740	727	$47,379	39,669	16,086	7.0	$ 12,107	22,405	$194,748,360	5.3
50-54	20,649	$11,412	21,058	1,333	$46,776	45,604	21,982	9.5	$ 13,556	24,775	$297,990,983	8.1
55-59	25,875	$12,440	20,838	1,989	$43,310	39,549	27,864	12.1	$ 14,643	24,042	$408,020,598	11.1
60-64	29,421	$13,556	21,560	2,733	$41,385	37,647	32,154	14.0	$ 15,921	24,617	$511,938,583	14.0
65-69	29,825	$14,908	21,668	3,689	$38,607	37,331	33,514	14.5	$ 17,517	25,024	$587,068,806	16.0
70-74	22,372	$16,575	21,687	4,093	$35,329	37,131	26,465	11.5	$ 19,475	25,628	$515,413,796	14.1
75-79	15,058	$17,446	21,139	4,073	$30,082	32,582	19,131	8.3	$ 20,137	24,586	$385,233,265	10.5
80-84	8,244	$17,608	20,742	3,497	$24,276	28,901	11,741	5.1	$ 19,597	23,667	$230,089,549	6.3
85-89	3,738	$16,355	20,904	2,424	$18,942	28,134	6,162	2.7	$ 17,373	24,041	$107,051,153	2.9
90+	1,280	$14,493	20,372	1,297	$12,810	19,144	2,577	1.1	$ 13,646	19,777	$35,166,203	1.0
**Total**	**203,495**	**$13,509**	**21,899**	**26,885**	**$33,912**	**37,064**	**230,380**	**100**	**$ 15,890**	**25,036**	**$3,660,694,073**	**100**

Table S2. Mean Annual Healthcare Costs (2013-16) in AU$ per Person by Type of Cancer and Health Service Component (Patient-level).

**Type of cancer (ICD-O3 code)**	**Total N**	**Mean cost per person**	**SD**	**Hospitalisations (public)**				**Pharmaceuticals**				**Medicare Services**				**Emergency Admissions**			
				**N**	**%**	**Mean cost**	**SD**	**N**	**%**	**Mean cost**	**SD**	**N**	**%**	**Mean cost**	**SD**	**N**	**%**	**Mean cost**	**SD**
Esophageal (C15)	1,200	$26,268	35,401	801	66.8	$26,062	39,180	1,172	97.7	$1,948	2,759	1,139	94.9	$ 6,208	7,184	740	61.7	$1,747	1,510
Stomach (C16)	1,941	$23,616	27,542	1,240	63.9	$24,214	28,950	1,890	97.4	$2,590	5,775	1,841	94.8	$ 4,805	5,869	1,185	61.1	$1,749	1,568
Colorectal (C18-20)	26,646	$17,858	25,155	14,452	54.2	$18,659	26,744	26,139	98.1	$3,123	7,322	25,790	96.8	$ 4,022	4,559	13,822	51.9	$1,508	1,372
Pancreatic (C25)	1,819	$27,185	31,554	1,221	67.1	$24,513	33,950	1,752	96.3	$4,262	6,361	1,693	93.1	$ 5,880	6,745	1,127	62.0	$1,861	1,721
Lung (C33-34)	8,750	$26,651	27,018	6,459	73.8	$23,119	26,291	8,390	95.9	$3,559	5,992	8,140	93.0	$ 5,210	5,850	5,798	66.3	$2,001	1,793
Melanoma (C44, M872-M879)	40,655	$9,487	19,092	14,847	36.5	$12,126	22,495	39,327	96.7	$2,012	8,005	39,876	98.1	$ 2,676	3,107	16,096	39.6	$1,232	1,136
Breast (C50)	37,745	$13,140	18,244	16,390	43.4	$13,057	19,403	37,170	98.5	$3,011	6,217	37,165	98.5	$ 4,011	4,125	16,318	43.2	$1,285	1,181
Cervical (C53)	2,266	$12,433	19,912	1,093	48.2	$16,338	21,860	2,181	96.2	$1,185	2,937	2,227	98.3	$ 2,762	4,068	1,103	48.7	$1,434	1,370
Uterine (C54)	4,893	$11,988	17,882	2,239	45.8	$14,785	21,049	4,808	98.3	$1,471	2,805	4,811	98.3	$ 3,205	3,568	2,204	45.0	$1,387	1,267
Ovarian (C56)	1,997	$18,710	23,649	1,007	50.4	$19,158	24,968	1,940	97.1	$3,540	5,397	1,932	96.7	$ 4,916	5,625	1,000	50.1	$1,706	4,740
Prostate (C61)	40,988	$13,119	19,470	18,399	44.9	$13,985	22,676	40,446	98.7	$2,528	5,391	39,833	97.2	$ 3,824	3,794	18,477	45.1	$1,398	1,275
Kidney (C64-66, C68)	6,016	$16,470	23,377	3,248	54.0	$17,082	24,659	5,893	98.0	$3,016	6,760	5,841	97.1	$ 3,568	3,919	3,234	53.8	$1,543	1,481
Bladder (C67)	3,574	$18,856	27,765	2,193	61.4	$19,661	31,315	3,520	98.5	$1,861	3,443	3,389	94.8	$ 4,230	4,899	2,029	56.8	$1,669	1,529
Brain (C70-72)	1,849	$30,264	37,161	1,199	64.8	$29,873	38,923	1,790	96.8	$3,863	5,382	1,800	97.3	$ 6,114	7,311	1,201	65.0	$1,849	1,580
Thyroid (C73)	6,098	$8,801	15,276	2,483	40.7	$11,522	19,376	6,026	98.8	$1,110	3,013	6,019	98.7	$ 2,622	2,883	2,362	38.7	$1,097	935
Non-Hodgkin Lymphoma (M959, M967-971)	7,700	$24,397	33,634	3,947	51.3	$22,573	34,981	7,533	97.8	$7,063	10,528	7,492	97.3	$ 5,250	7,326	3,953	51.3	$1,573	1,485
Leukemia (M980-M994)	6,308	$29,158	44,390	3,395	53.8	$28,543	47,525	6,131	97.2	$8,273	15,130	6,119	97.0	$ 4,951	7,601	3,470	55.0	$1,732	1,582
Myeloma (M973)	2,201	$45,951	39,499	1,398	63.5	$28,057	33,187	2,170	98.6	$19,657	20,772	2,143	97.4	$ 7,914	8,986	1,282	58.2	$1,793	1,585

Unknown primary site (C80)	2,303	$20,651	26,491	1,424	61.8	$19,179	23,214	2,120	92.1	$4,252	13,318	2,034	88.3	$ 4,499	5,081	1,282	55.7	$1,626	1,429
Head & neck (C01-14, C30-32)	6,323	$20,525	29,892	4,220	66.7	$20,861	31,665	6,173	97.6	$1,907	4,321	6,136	97.0	$ 3,985	4,175	3,576	56.6	$1,545	1,382
Liver (C22)	1,342	$29,619	37,306	1,038	77.3	$24,878	35,798	1,266	94.3	$6,036	13,723	1,241	92.5	$ 3,609	4,459	920	68.6	$1,962	1,881
Other	17,766	$17,739	31,207	9,127	51.4	$20,092	35,212	17,163	96.6	$3,185	7,896	17,281	97.3	$ 3,628	5,082	9,241	52.0	$1,560	1,510
**Total**	**230,380**	**$ 15,890**	**25,036**	**111,820**	**48.6**	**$17,297**	**27,542**	**225,000**	**97.7**	**$3,127**	**7,746**	**223,942**	**97.2**	**$ 3,843**	**4,635**	**110,420**	**47.9**	**$1,471**	**1,448**
